# Study on SARS-CoV-2 transmission and the effects of control measures in China

**DOI:** 10.1371/journal.pone.0242649

**Published:** 2020-11-30

**Authors:** Bo Zhang, Hongwei Zhou, Fang Zhou

**Affiliations:** 1 School of Environment and Natural Resources, Renmin University of China, Haidian District, Beijing, China; 2 Department of Ophthalmology, Lianshui County People’s Hospital, Huai’an, Jiangsu, China; 3 School of Medicine, Southeast University, Nanjing, Jiangsu, China; 4 Beijing Key Laboratory of Megaregions Sustainable Development Modeling, Capital University of Economics and Business, Fengtai District, Beijing, China; 5 College of Urban Economics and Public Administration, Capital University of Economics and Business, Fengtai District, Beijing, China; The University of Hong Kong, CHINA

## Abstract

**Objective:**

To reconstruct the transmission trajectory of SARS-CoV-2 and analyze the effects of control measures in China.

**Methods:**

Python 3.7.1 was used to write a SEIR class to model the epidemic procedure and proportional estimation method to estimate the initial true infected number. The epidemic area in China was divided into three parts, Wuhan city, Hubei province (except Wuhan) and China (except Hubei) based on the different transmission pattern. A testing capacity limitation factor for medical resources was imposed to model the number of infected but not quarantined individuals. Baidu migration data were used to assess the number of infected individuals who migrated from Wuhan to other areas.

**Results:**

Basic reproduction number, R_0_, was 3.6 before the city was lockdown on Jan 23, 2020. The actual infected number the model predicted was 4508 in Wuhan before Jan 23, 2020. By January 22 2020, it was estimated that 1764 infected cases migrated from Wuhan to other cities in Hubei province. Effective reproductive number, R, gradually decreased from 3.6 (Wuhan), 3.4 (Hubei except Wuhan,) and 3.3 (China except Hubei) in stage 1 (from Dec 08, 2019 to Jan 22, 2020) to 0.67 (Wuhan), 0.59 (Hubei except Wuhan) and 0.63 (China except Hubei) respectively. Especially after January 23, 2020 when Wuhan City was closed, the infected number showed a turning point in Wuhan. By early April, there would be 42073 (95% confidence interval, 41673 to 42475), 21342 (95% confidence interval, 21057 to 21629) and 13384 (95% confidence interval, 13158 to 13612) infected cases in Wuhan, Hubei (except Wuhan) and China (except Hubei), respectively.

**Conclusion:**

A series of control measures in China have effectively prevented the spread of COVID-19, and the epidemic should be under control in early April with very few new cases occasionally reported.

## Introduction

On December 31, 2019, Hubei Provincial Health Commission announced the discovery of some clusters of pneumonia without definite cause [1]. Sequences of 2 complete viral genomes of 29.8 kilobases (HKU-SZ-002a and HKU-SZ-005b) obtained from 2 patients with the pneumonia were identified, and a novel lineage B coronavirus closely related to bat SARS-like bat-SL-CoVZXC21 (NCBI accession number MG772934) and bat-SL-CoVZC45 (NCBI accession number MG772933) was revealed [1]. The World Health Organization (WHO) temporarily named the novel coronavirus as 2019-nCoV, and recommended that the novel coronavirus infected pneumonia be named 2019-nCoV acute respiratory disease. On Feb 11, 2020, the WHO announced that 2019-nCoV infection has finally been given an official name COVID-19, and the Coronavirus Study Group (CSG) of the International Committee on Taxonomy of Viruses had named 2019-nCoV severe acute respiratory syndrome-related coronavirus 2, or SARS-CoV-2 [2]. It is believed that human infection with SARS-CoV-2 first occurred in Wuhan on November 9, 2019 (95% credible interval: 25 September 2019 and 19 December 2019) [3]. 27 cases were initially reported, and 41 cases were reported by January 11, 2020, including 7 severe cases and 1 death [1].

SARS-CoV-2 infected patients typically clinically manifested with fever, respiratory symptoms (cough, breathing difficulties); radiographic ground-glass lung changes; normal or below-average white blood cell, lymphocytes and platelet counts; hypoxemia; and liver and kidney dysfunction. It is reported that 5/41 cases can be combined with virus-related cardiac injury [4], and SARS-CoV-2 infected patients can have gastrointestinal symptoms [1, 5].

Some of the initial cases of severe acute respiratory syndrome coronavirus 2 (SARS-CoV-2) infection were geographically related to the Huanan Seafood Wholesale Market [6]. On January 24, 2020, Chan JF et al. reported the existence of human-to-human transmission of SARS-CoV-2 [1]. A total of 425 laboratory-confirmed cases were reported by January 22, 2020, including 5 clusters comprising 16 cases by January 16, 2020, and some cases were found to have no apparent history of exposure [6]. In addition, a case of asymptomatic contact infection was reported by German researchers, which was the first confirmation that latent cases can be contagious [7].

In the early stage of the COVID-19 outbreak, the mean doubling time was estimated to be 7.4 days (95% confidence interval: 4.2 to 14), the basic reproductive number R_0_ was estimated to be 2.2 (95% confidence interval: 1.4 to 3.9), the mean incubation period was estimated to be 5.2 days (95% confidence interval: 4.1 to 7.0), and the serial interval distribution was estimated to have a mean (±SD) of 7.5 ± 3.4 days (95% confidence interval: 5.3 to 19) [6]. By January 29, 2020, the mortality rate of COVID-19 was approximately 2.1%, similar to the overall figures currently available [8].

Humans have always been fighting against infectious diseases. Humans will take a series of practical prevention and control measures to prevent the spread of infectious diseases. When epidemic prevention measures (e.g., isolation, blockades, etc.) are taken, infectious diseases cannot spread under ideal circumstances. The effective reproductive number, R, refers to the size of secondary cases infected by one infected case during the actual spread of an infectious disease. In the absence of control measures, R = R_0_x, where x refers to the proportion of susceptible people. In the process of infectious disease transmission, R will be reduced due to the implementation of special control measures and a reduction in the number of susceptible individuals [9].

The diagnostic criteria for COVID-19 are described as follows. The American Centers for Disease Control and Prevention (CDC) combined epidemiological risk factors (travel from the city of Wuhan, close contact with a person who is under investigation for COVID-19 while that person was ill or close contact with an ill individual with laboratory-confirmed COVID-19 in the last 14 days before illness onset) and clinical features (fever, symptoms of lower respiratory illness) to diagnose suspected COVID-19 [10]. The definite diagnosis of COVID-19 can be make if the 344bp RNA-dependent RNA poly-merase (RdRp) gene related to severe acute respiratory syndrome (SARS) and 158bp spike (s) gene specific to SARS-CoV-2 were detected by real-time reverse-transcription–polymerasechain-reaction (RT-PCR) [1], or if two targets (open reading frame 1a or 1b, nucleocapsid protein) were detected by RT-PCR [6]. Diagnostic criteria of COVID-19 of the Chinese Center for Disease Control and Prevention (CDC) is as follow. Suspected or probable cases are defined as cases that meet: (1) three clinical standards or (2) two clinical standards and one epidemiological criteria. Clinical criteria are: fever; radiographic evidence of pneumonia or acute respiratory distress syndrome; low or normal white blood cell count, or low lymphocyte count. Epidemiological criteria are: living in Wuhan or have a history of travel to Wuhan within 14 days before the onset of symptoms; contact with patients with fever and respiratory infection symptoms within 14 days before the onset of symptoms; and a link to any confirmed cases or clusters of suspected cases [11]. For the first case in a province, a confirmed case is defined as a suspected or probable case with positive viral nucleic acid detected at the municipal and provincial CDC. For the second case and all subsequent cases, it is defined as suspected or probable cases with positive viral nucleic acid detected at the municipal CDC [11].

SARS-CoV-2 is a novel virus previously unknown. Numerous studies have revealed its genetic sequence and source. A team led by Professor Zhang YZ from Shanghai Public Health Clinical Center released the first SARS-CoV-2 gene sequence on the website of viral.org on January 11, 2020 [12]. On January 12, 2020, five other virus genome sequences from different patients were released by the National Health Commission (NHC) of the People’s Republic of China in Global Initiative of Sharing All Influenza Data (GISAID), a global shared influenza virus database [13]. As of January 19, 2020, GISAID (http://gisaid.org/) has released 13 SARS-CoV-2 genome sequences. One virus strain (Virus name: BetaCoV/Wuhan-Hu-1/2019; Accession ID: EPI_ISL_402125) has been released on GenBank (https://www.ncbi.nlm.nih.gov/nuccore/MN908947) by the Shanghai Public Health Clinical Center. Meanwhile, Chan JF et al. Constructed phylogenetic trees by genetic analysis of RdRp and S gene sequences of PCR amplified fragments from five patients, and revealed that SARS-CoV-2 is closely related to bat SARS-like bat-SL-CoVZXC21 (NCBI accession number MG772934) and bat-SL-CoVZC45 (NCBI accession number MG772933) [1]. On January 23, 2020, a team led by Shi ZL form Wuhan Institute of Virology published an article revealed that genome of SARS-CoV-2 has 96.2% similarity compared to that of RaTG13, a bat coronavirus previously detected on the Rhinolophus sinicus in Yunnan province and 79.5% similarity compared to that of SARS coronavirus [14]. And one study has suggested that the intermediate host of SARS-CoV-2 may be snake [15].

It was suggested that human-to-human transmission may have occurred in Wuhan in the early stage of outbreak. Based on the data analysis of COVID-19 incidence in Wuhan from December 10, 2019 to January 4, 2020, it was shown that although most of the early COVID-19 had a history of Huanan Seafood Wholesale Market exposure, since the end of December 2019, the number of cases without a history of exposure had grown exponentially [6]. And from January 13, 2020 when Wuhan officially announced the outbreak of COVID-19 to January 21, 2020, the sudden decrease in the number of cases should be due to the underestimation of the number of cases and the delay in the confirmation report [6]. Several studies have estimated the transmission dynamics parameters of COVID-19. In the early stage of COVID-19 outbreak, basic reproductive number R_0_ was estimated to be 2.2 (95% confidence interval, 1.4 to 3.9) [6]. Of the 45 people who became ill before 1 January, the average duration from illness onset to first medical visit was 5.8 days (95% confidence interval, 4.3 to 7.5); of the 44 people who became ill before 1 January, the average interval from the disease onset to hospitalization was 12.5 days (95% confidence interval, 10.3 to 14.8) [6]. A study by the University of Hong Kong estimated that the basic reproductive number R_0_ was 2.68 and the doubling time of the number of cases was 6.4 days [11].

After the outbreak of COVID-19, the Chinese government and people quickly took measures to control the sources of infection and block the routes of transmission, and the spread of the epidemic was effectively curbed. However, the lack of proper diagnostic tools, the focus on the more severe cases and the overcrowding of hospitals made the reporting rate significantly lower in the city of Wuhan than elsewhere. In this study, mainly based on the Susceptible-Exposed-Infected-Removed (SEIR) model, we studied the transmission dynamics of SARS-CoV-2 under various practical situations. In this study, we divided the epidemic area into three parts, imposed the testing capacity limitation factor of medical resource to model the infected but not quarantined especially before Jan 23, 2020, and a proportional estimation method was used to estimate the number of potential infections which is much larger than the official number announced. We strived to reconstruct the transmission trajectory of SARS-CoV-2 in China and analyze the effects of China’s control measures.

## Materials and methods

Our methodology was mainly based on the Susceptible-Exposed-Infected-Removed (SEIR) model, which is a very popular method when there is a considerable post-infection incubation period. The epidemic area was divided into three parts, Wuhan, Hubei (except Wuhan) and China (except Hubei) based on the different transmission patterns. A testing capacity limitation factor for the medical resource θ was imposed to model which stands for the maximum testing capacity per day. Since the first case was diagnosed on 8 Dec 2019, a large number of infected people could not be quarantined due to testing capacity limitations. As a result, the model shows that the number of potential infections was much larger than that officially announced before Jan 23, 2020.

Python 3.7.1 was used to write a SEIR class to model the epidemic procedure and proportional estimation method to estimate the initial infected number and other parameters.

The authors had not access to information that could identify individual participants during or after data collection.

### Susceptible-exposed-infected-removed model

The classical SEIR models the number of people in the four states: susceptible (S), exposed (E), infected (I) and removed (R). In this study, we imposed a new state quarantine (Q) to model the number of quarantine which was high related with the testing capacity and medical resources. Since the number of infected could not be observed directly, Q could be regard as announced infected number because anyone who was tested and confirmed would be quarantined according to epidemic prevention policy. A parameter β controls how fast people move from S to E where β = k × b. Parameter k stands for an average number of people one contacts per day and parameter b stands for the infection probability when susceptible people contacted an exposed one who has been infected. Many government prevention and control measures affect k and b to slow down the spread of the epidemic, such as home isolation aims to reduce the number of people one contacted and going out with a mask aims to reduce the infection probability when contacted with infected ones. The parameter σ stands for the reciprocal of the duration that from infection to diagnosis which controls the speed from state E to state I. Big σ means people could be tested or confirmed very soon when they infected, and they would have much fewer chances to infect others. Parameter γ stands for reciprocal of the duration from diagnosis to recovery which controls the speed from state I to state R. Parameter θ stands for the maximum capacity of testing. The dynamic procedure of epidemic can be described by the following equation:

{dSdt=-βSINdEdt=βSIN-σEdIdt=-γI-QdQdt=min(θ,I)dRdt=γI

Due to the limited testing capacity, many people could not be confirmed although they had been already infected, it meant they could still infected others until the testing result was positive and been isolated. In our model we used min(*θ*, *I*) to model the maximum capability of testing that infected people could be isolated timely. If the number of infection was greater than the testing capacity limitation factor (where *I* > *θ*), only θ number of infected people could be isolated timely or diagnosed at time point t and the others still could infect others, which would result in the infection number announced is less than the overall infection number. As the capacity increase, there would be little or no limitation for the testing capacity and anyone who was tested positive could be isolated timely, the infection number announced would get closer to the actual number. There could be big gap between the infected number and announced number when the testing and isolation capacity was very limited. From the formula above, we start the dynamic process with S_1_ = *N* − *I*_1_, *E*_1_ = 1, *I*_1_ = 1, R_1_ = 0 with parameters σ = 1/10, *γ* = 1/14, the only unknown parameter was β. We simulated the model by changed the β from 0 to 1 step with 0.0001. The best β was choose when it minimized the absolute difference between the simulated I_t_ and I_t_. We could get R_0_ by the following formula R_0_ = *kb*/*γ* = β/*γ* [9]. Another indicator that concerned was death rate which helped to estimate the total death for the epidemic. Death rate was affected by many factors such as medical level and individual characters and so on, it was very hard to figure out the mechanism for each factor. But total death rate increased gradually because some infected people may die even though there was no new added infected. We used a log function to fit the death rate based on the infected number we estimated and death number official announced.

### Estimation of the migration by Baidu migration

Baidu migration (https://qianxi.baidu.com/) data was collected by Baidu Map when people use the navigation system. To protect user privacy, Baidu migration only provided the proportion data that migrated in and out from one city to another. In the study, we collection the migration proportion data from Wuhan to other cities from Jan 14, 2020 to Jan 23, 2020, then summarized the migration proportion by provinces and the top 8 provinces were Henan, Hunan, Guangdong, Anhui, Jiangxi, Chongqing, Beijing and Shanghai. About 8% of the total migration from Wuhan were flowed into these 8 provinces. 5 million people left Wuhan according to government press conference from Jan 10 2020 [16], some of them had been infected already and took the virus across the country. We multiplied the migration proportion with total migration number and found about 400 thousand people had flowed into these provinces before Wuhan was lockdown.

### Estimation of initial values

The model is highly dependent on the initial values of S, E, I, R and other parameters, better parameters was the key to better results. It is well known that the actual number of infections were often underestimated and were much greater than the reported number especially in the early days of outbreak. In this study, we used a proportional estimation method to estimate the actual number of infections in Wuhan based on the number of total population and migrants from Wuhan to other cities based on the assumption that the infected proportion in Wuhan was equal to the migration people from Wuhan. Because before Jan 23, 2020, government took no control actions and the infected proportion was uniform distributed in the population. Although there have been several reports of incubation time [1, 6, 10]. Chan JF et al. [1] estimated that the incubation period of COVID-19 was 3 to 6 days. Li Q’s study demonstrated the incubation period of 5.2 days (95% confidence interval [CI], 4.1 to 7.0), with the 95th percentile of the distribution at 12.5 days.) [6]. Carlos WG [10] reported that the incubation period appears to range between two days to up to two weeks following exposure. There is no exact conclusion. And the diagnosis time may be about 3 days later than the onset time. We assumed that incubation period could be 7 days and 3 days more needed to diagnosis which means the duration from E to I could be 10 days. We collected the provincial infected data from the database (http://2019ncov.chinacdc.cn/2019-nCoV/) developed by the Chinese Center for Disease and Prevention (China CDC). We summed the infected number from the top 8 provinces and obtained 1568, and we marked this number with a parameter m and the migration number of top 8 provinces from Wuhan as parameter M. We used N as the total population of Wuhan which was 11.5 million from Wuhan Statistical Yearbook 2018. Parameter $\hat{e}$ denoted for the actual number of infections in Wuhan on Jan 23, 2020. We used formula e^=mM*N to estimate the actual infected number in Wuhan on Jan 22, 2020. The result showed there might be 4508 infected people which was 10 times more than official announced number of 425 [6].

Since the first case was reported at Dec 8, 2019 and we used the date as the start time of epidemic. In the model, we assumed parameter k was equal to 5 meant one could contacted five people per day on average. There were 45 days from Dec 8, 2019 to Jan 22, 2020 and I_45_ = 4508, then we simulated the process and got parameter β was 0.2584, parameter b was 5% and the basic reproduction number (R_0_) was estimated equal to 3.6 before Jan 23, 2020 in Wuhan.

For Hubei province (except Wuhan), Baidu migration data showed that about 90% population left Wuhan (about 4.5 million) were flowed into other cities in Hubei province. We estimated the number of infections would be 1764 on Jan 22, 2020 based on the same proportional estimation method, and R_0_ for Hubei was 3.4 by optimal fitted the infected curve and the probability of being infected after each contact was 5% the same as Wuhan.

## Results

### Outcomes about Wuhan

**Fig 1:**
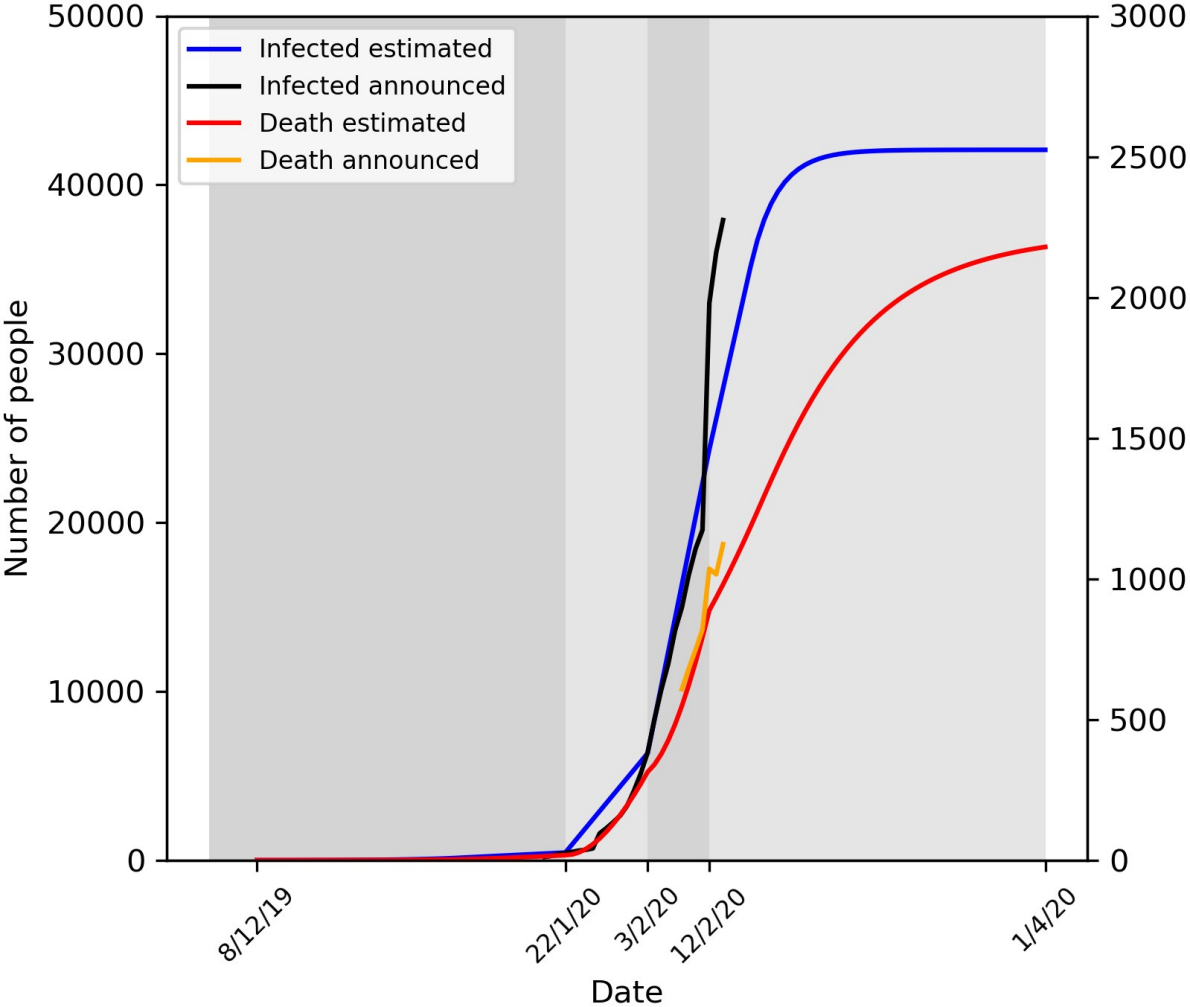
The predict number of infected and death in Wuhan in the four stages.

The epidemic started from Wuhan and showed four significantly different stages (Fig 1). Stage 1 started from Dec 8, 2019 to Jan 22, 2020. In this stage, most people were not aware of the severity of the epidemic and government did not take effective measures. The infected number was much more than official announced and could be 4508 according to our model. These infected people were not treated effectively and isolated, which led to the rapid spread of the epidemic. Stage 2 started from Jan 23, 2020 to Feb 3, 2020, Wuhan was completely closed, everybody was required to stay at home to avoid to contact with others, and which leading the infected number growth curve changed from exponential to nearly linear. Because most people stayed at home and wear mask when going out, the infected probability was reduced to 3%. But the testing capacity is very limited and not everyone could be diagnosed timely. And only confirmed cases could be isolated treated in hospitals. Stage 3 started from Feb 4, 2020 to Feb 12, 2020. In the third stage, central government decided to deploy resources from the whole country to Wuhan [17, 18], leading to the testing capacity greatly increased from hundreds per day to 24000 per day and hospital beds increased 13000 in Wuhan [19]. The infected probability was reduced to below 2% and anyone who had positive testing result could be isolated and treated in hospitals and the average duration from status E to I was reduced from 10 days to 5 days which means people could be treated more faster and he had little chances to infect others. The infected number showed a turning point and started to decline gradually. Stage 4 started from Feb 13, 2020, China’s pragmatic approach worked and the testing capacity and hospital beds could meet all the demand [20], the infected number declined rapidly. Finally, the epidemic would be basically under control in early April with very few new cases. On Apr 1, 2020, the model predicted there would be 42073 (95% confidence interval, 41673 to 42475) infected and 2179 (95% confidence interval, 2088 to 2270) death in Wuhan. The death rate was 3.9%, 4.9%, 3.6% and 5.1% respectively in each stage. In last stage, the death rate was slightly higher than other stage, because the treatment would last for a long time for the critically ill patients.

Key parameters and results in Wuhan prediction model were shown in Table 1.

**Table 1:** Key parameters and results in Wuhan prediction model.

	Duration	R	Limitation θ	Infected estimated	Infected announced	Death estimated	Death announced
Stage 1	Dec 8, 2019-Jan 22, 2020	3.6	100 per day	453	425	18	17
Stage 2	Jan 23, 2020-Feb 3, 2020	1.55	500 per day	6333	6384	313	313
Stage 3	Feb 4, 2020-Feb 12, 2020	1.08	2000 per day	24333	32994	1028	1036
Stage 4	Feb 13, 2020-Apr 1, 2020	0.67	unlimited	42073	-	2179	-

### Outcomes about Hubei (except Wuhan)

**Fig 2:**
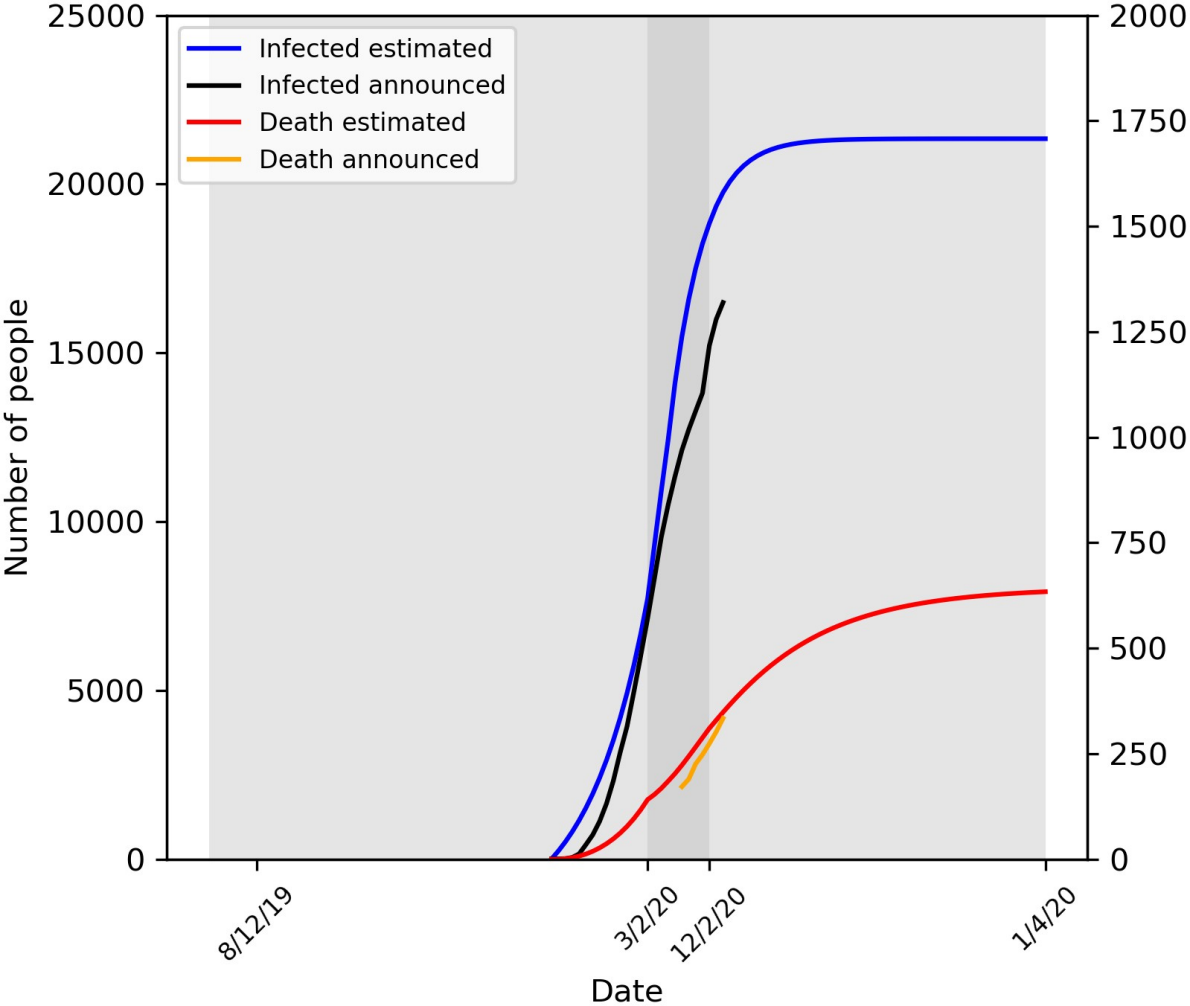
The predict number of infected and death in Hubei (except Wuhan) in the three stages.

The epidemic in Hubei (except Wuhan) showed three significantly different stages (Fig 2). According to the official announcement, about 5 million people left Wuhan from Jan 10, 2020 to Jan 22 2020 [16], it means about 4.5 million people migrated into Hubei and about 1764 infected among them we estimated. Stage 1 started from Jan 23, 2020 to Feb 3, 2020, the infected number increased exponentially because many infected people came from Wuhan for the Spring Festival holiday who infected more people and the R_0_ was 3.4 in this stage. On Feb 3, 2020, we estimated there would be 15122 infected about 2 times more than official announced 7138. It means a lot of infected were not be tested. Stage 2 started from Feb 4, 2020 to Feb 12, 2020, the added infected number reached the peak as all the cities in Hubei took strict control measures such as closed all the highway, railway and airplane to other provinces or overseas and the testing capacity was greatly increased with assistance from other provinces. The infected probability was reduced to 1.6% and duration from infected to isolation in hospital was reduced to 5 days on average. R_0_ was reduced to below 1 which means the epidemic was under controlled. Stage 3 started from Feb 13, 2020 to Apr 1, 2020, the added infected number decreased significantly. On Apr 1, 2020 there would be 21342 (95% confidence interval, 21057 to 21629) infected and 633 (95% confidence interval, 585 to 683) death in Hubei (except Wuhan). The death rate for Hubei would be 2.9% and much lower than Wuhan because the infected was distributed in more cities and there was much more medical resources for each patient.

Key parameters and results in Hubei (except Wuhan) prediction model were shown in Table 2.

**Table 2:** Key parameters and results in Hubei (except Wuhan) prediction model.

	Duration	R	Limitation θ	infected estimated	Infected announced	death estimated	Death announced
Stage 1	Jan 23, 2020-Feb 3 2020	3.4	1000 per day	7719	7138	141	101
Stage 2	Feb 3, 2020-Feb 12, 2020	0.83	2000 per day	18832	15212	310	274
Stage 3	Feb 13 2020-Apr 1 2020	0.59	unlimited	21342	-	633	-

### Outcomes about China (except Hubei)

**Fig 3:**
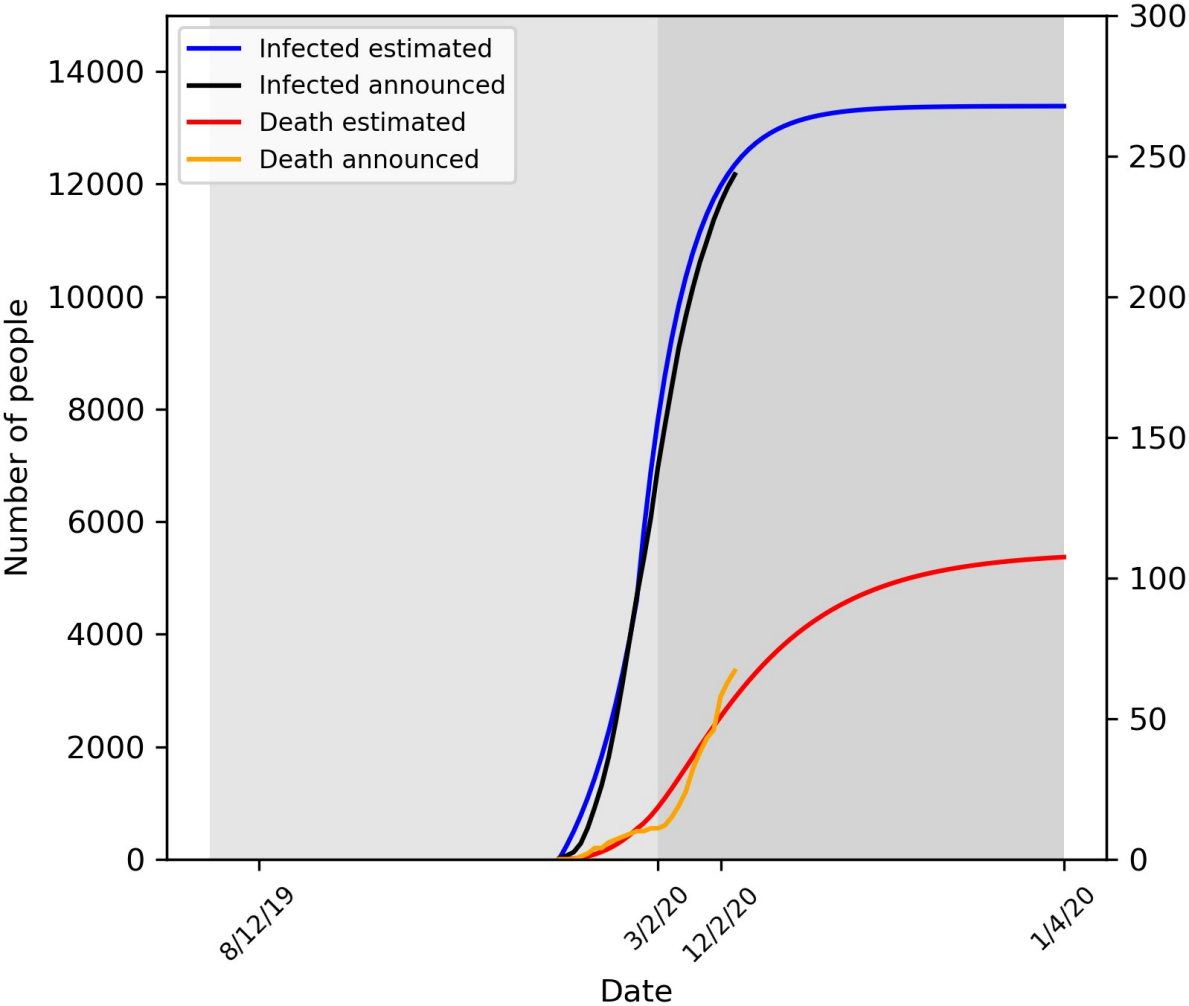
The predict number of infected and death in China (except Hubei) in the two stages.

The epidemic transmission process could be divided into two stages (Fig 3). In Stage one (from Jan 23, 2020 to Feb 3, 2020), the infected number increased rapidly. The initial R_0_ for China (except Hubei) was 3.3 only slightly lower than that of Wuhan. Because the infected number was much lower than Wuhan and anyone who migrated from Wuhan or Hubei was asked to isolated himself at home. The infected number was estimated about 9902 by our model which was 42% more than the official announced 6949 infected number. In stage two (from Feb 4, 2020 to Apr 1, 2020), the infected number got its peek around February 3, 2020. It was estimated that the total infected and death number in China (except Hubei) could be 13384 (95% confidence interval, 13158 to 13612) and 107 (95% confidence interval, 87 to 128). The death rate was only 0.8% about one third of Hubei and one fifth of Wuhan.

Key parameters and results in China (except Hubei) prediction model were shown in Table 3.

**Table 3:** Key parameters and results in China (except Hubei) prediction model.

	Duration	R	Limitation θ	infected estimated	Infected announced	death estimated	Death announced
Stage 1	Jan 23, 2020-Feb 3, 2020	3.3	unlimited	7793	6949	18	11
Stage 2	Feb 4, 2020-Apr 1, 2020	0.63	unlimited	13384	-	107	-

## Discussion

In the early stage of COVID-19 outbreak, the medical resources in Wuhan City were in short supply; the hospitals were overcrowded; the number of hospital beds and medical staff is insufficient; the corresponding diagnostic reagents were lacking; and the uniform diagnostic standards were missing. Some people don’t pay much attention to COVID-19, and some SARS-CoV-2 infected patients with mild illness do not go to hospital. That all made the SARS-CoV-2 infected cases could not be accurately diagnosed in time. Local administrators and health management officials were not aware of the severity of the SARS-CoV-2 epidemic. They were afraid to take responsibility, so they may cover up the number of cases deliberately and report them late. The reporting rate may be significantly lower in Wuhan city in early stage due to previously described reasons. The WHO Collaborating Centre for Infectious disease modelling estimated that the number of SARS-CoV-2 infected cases in Wuhan by 18 January, 2020 was approximately 4000 (95% confidence interval: 1000–9700) [21]. The number is much larger than officially announced. Hiroshi Nishiura et al. through a study on the number of cases exported from Wuhan and the spatial back-calculation method, suggested that by January 24, 2020, the cumulative number of cases in China was about 5502 (95% confidence interval: 3027, 9057) [22]. As we all know, the emergence of super-spreaders is very important for the rapid spread of SARS-CoV-2 in the early stage. In view of the event of one patient spread the virus to fourteen medical personnel, it is suggested that epidemic super-spreaders were generated in the early stage of virus transmission from animals to humans and in the early stage of transmission outbreak [3]. Therefore, in the early transmission stage of COVID-19, the number of cases reported by Wuhan officially should be lower than the actual number of cases in Wuhan.

In this study, we built SEIR models with a medical resource limitation factor that modeled the maximum number of people who could be treated and isolated. We used a proportional estimation method to estimate the number of potential infections and divided the epidemic area into three parts, Wuhan, Hubei (except Wuhan) and China (except Hubei), based on the different transmission patterns. In our study, we found that SARS-CoV-2 has an R_0_ value of 3.6, the spread of SARS-CoV-2 in China was effectively suppressed, and the epidemic should be under control in early April with very few new cases occasionally reported.

Chinese People, governments at all levels have taken active measures to control the spread of COVID-19. According to our research, these measures effectively controlled the spread of COVID-19. The following measures have important reference significances for the control of infectious diseases may occur in the future. On December 31, 2019, the NHC expert group arrived in Wuhan to carry out the relevant testing and verification work [23]. On January 01, 2020, Wuhan City shut down the Huanan Seafood Wholesale Market [24]. On January 07, 2020, the NHC expert group initially identified the pathogen in these unexplained cases of viral pneumonia as a novel coronavirus [23]. On January 20, 2020, Chinese government issued important instructions saying that the recent outbreak of pneumonia with novel coronavirus infection in Wuhan City, Hubei Province must be given great attention and all efforts to prevent and control [23]. On January 21, 2020, The NHC issued 2020 No. 1 Proclamation to incorporate the novel coronavirus pneumonia into the Class B infectious diseases and take the prevention and control measures of Class A infectious diseases [25, 26]. At 10 a.m. on January 23, 2020, Wuhan City Bus, Subway, Ferry and Long-distance Passenger Transport was suspended, and the airport, railway station to leave Wuhan was temporarily closed [23]. On January 24, 2020, Hubei Province launched the major public health emergency Level I response [27], and then as of January 25, 2020, a total of 30 provinces (regions, cities) in the country launched the major public health emergency I-level response [28]. On January 27, 2020, the General Office of the State Council extended the Spring Festival holiday to February 2 [26]. On January 27, 2020, the NHC issued the " Guidelines for the Diagnosis and Treatment of Novel Coronavirus (2019-nCoV) Infection by the National Health Commission (Trial Version 4)" [23]. On February 4, 2020, a designated makeshift infirmary named Wuhan Fire God Hill Hospital began to officially receive confirmed cases [26]. On February 8, 2020, another designated makeshift infirmary Thunder God Hill Hospital was delivered to receive confirmed cases [26]. On February 5, 2020, three "mobile field hospitals" began to be built in Wuhan [29, 30]. By February 8, 2020, central government of China has mobilized some 11000 medical personnel to support Wuhan [31]. On February 9, 2020, Wuhan city took measures to try to collect all the suspected cases and confirmed cases of mild illness into the hospital for centralized treatment [32]. On 9 February 2020, another group of medical personnel of some 6,000 people entered Wuhan to provide medical support [33].

Meanwhile, China’s scientific research institutions and medical equipment manufacturing enterprises quickly took measures to speed up scientific research and production of medical supplies. Scientific research institutions in China have done a lot of in-depth researches on the source, sequencing of SARS-CoV-2, disease transmission dynamics, clinical manifestations, drug treatment, diagnostic standards, diagnostic reagents of COVID-19, etc. On January 26, 2020, the China National Medical Products Administration (NMPA) approved four SARS-CoV-2 detection products to fully serve epidemic control needs [34]. On February 1, 2020, the daily output of Chinese SARS-CoV-2 test kits was 773,00, reaching a maximum production capacity of approximately 60% to 70% [35]. By March 8, 2020, the daily production of medical protective clothing in China had increased to 500,000 suits from the initial number of less than 20,000 suits [36].

In the first stage of SARS-CoV-2 transmission in Wuhan, the true number of infections was much higher than the officially announced probably because that people did not know COVID-19 is contagious and the local government did not take any control measure. In this stage, the infected cases were not effectively diagnosed, isolated, and treated, which led to rapid transmission of COVID-19. In the second stage, the trend of exponential growth of infection was suddenly controlled. The reason may be that Wuhan city was closed, people got the news that COVID-19 was contagious, and more and more medical resources were put into Wuhan. The medical capacity was still not sufficient to isolate every suspected or confirmed case and many infected people could not been treated or isolated timely. In the third stage, infected number growth curve in Wuhan showed a turning point and a gradual decline, possibly due to the input of medical resources and the strengthening of control measures. In the fourth stage, after February 13, 2020, the number of infected cases decreased rapidly, which is likely due to the substantial investment of medical resources and the strengthening of control measures, including the adoption of the new diagnostic standard "clinical diagnostic standard". In the first stage of SARS-CoV-2 transmission in Hubei (except Wuhan), the infected number increased exponentially should because of the infected people from Wuhan were not all quarantined and the medical resource were not sufficient. In this stage, the central government decided that one other province should support one city in Hubei. In the second stage, nearly all the infected and suspected people were quarantined, the added infected number reached the peak. The epidemic in China (except Hubei) was not so serious because of the sufficient medical resources and strong government control policy. In Stage one (from January 23, 2020 to February 3, 2020), the infected cases increased rapidly because of these virus carriers mainly infected his or her family members. In stage two (from February 4), most people canceled all their celebration activities and kept staying at home, and it greatly reduced the probability of infection. Anyone migrated from Hubei will be quarantined for at least 14 days. These dramatically reduced exposed number and the infected number got its peek around February 3, 2020.

The measures have effectively suppressed the spread of COVID-19. The infectious will spread sharply when R is greater than 1 and will gradually disappear when R is less than 1. Over time, R gradually decreased from 3.6 (Wuhan, stage 1), 3.4 (Hubei except Wuhan, stage 1) and 3.3 (China except Hubei, stage 1) to 0.67 (Wuhan, stage 4), 0.83 (Hubei except Wuhan, stage 2) and 0.63 (China except Hubei, stage 2), respectively. Especially after January 23, 2020 when Wuhan City was closed, the infected number showed a turning point in Wuhan in the next two weeks.

The outbreak of COVID-19 posed a major challenge to China’s Health system. Although the early human-to-human transmissions were described in the scientific literature, the local government of Hubei was not aware of the severity and did not inform the public timely, resulting in the public have no awareness of protection. Meanwhile, more than 5 million people left Wuhan with about 1960 infected among them during this time, and the outbreak spread across the country. At the very early stage of the outbreak of COVID-19, hospitals in Hubei Province had a serious shortage of medical protective equipment in routine reserves, which became the key limitation factor in controlling the outbreak. These defects should be improved in the future.

On February 12, 2020, the new diagnostic standard "clinical diagnostic standard" was adopted in Hubei province. Vast majority of new clinically diagnosed cases were diagnosed from accumulated suspected cases. Clinical diagnosis standard is different from the laboratory diagnosis standard widely used in the world. On February 12, 2020, Health Commission of Hubei Province announced that there were 14840 new confirmed cases in Hubei (including 13,332 clinically diagnosed cases). This does not mean that the epidemic trend has become worse. It is just a reflection of the change of diagnostic standards and the intensification of control measures. Data from Official of Hubei Province on February 12, 2020 were not used in this study, but could be used to evaluate some results of this study.

There were some limitations in this study, first the migrate data in the model was from Baidu migration data set which might not cover all the population migrated. Second, there was no accurate medical resource limitation published especially in Wuhan and we estimated the medical limitation by the maximum number of infected announced in the early stage and the added capacity of new hospitals in the later stage.

## Supporting information

S1 Data (XLSX)
